# Accelerated nonlinear interactions in graded-index multimode fibers

**DOI:** 10.1038/s41467-019-09687-9

**Published:** 2019-04-09

**Authors:** M. A. Eftekhar, Z. Sanjabi-Eznaveh, H. E. Lopez-Aviles, S. Benis, J. E. Antonio-Lopez, M. Kolesik, F. Wise, R. Amezcua-Correa, D. N. Christodoulides

**Affiliations:** 1 CREOL, College of Optics and Photonics, University of Central Florida, Orlando, FL 32816-2700 USA; 2 The College of Optical Sciences, The University of Arizona, Tucson, AZ 85721 USA; 3 School of Applied and Engineering Physics, Cornell University, Ithaca, NY 14853 USA

## Abstract

Multimode optical fibers have recently reemerged as a viable platform for addressing a number of long-standing issues associated with information bandwidth requirements and power-handling capabilities. As shown in recent studies, the complex nature of such heavily multimoded systems can be effectively exploited to observe altogether novel physical effects arising from spatiotemporal and intermodal linear and nonlinear processes. Here, we study for the first time, accelerated nonlinear intermodal interactions in core-diameter decreasing multimode fibers. We demonstrate that in the anomalous dispersion region, this spatiotemporal acceleration can lead to relatively blue-shifted multimode solitons and blue-drifting dispersive wave combs, while in the normal domain, to a notably flat and uniform supercontinuum, extending over 2.5 octaves. Our results pave the way towards a deeper understanding of the physics and complexity of nonlinear, heavily multimoded optical systems, and could lead to highly tunable optical sources with very high spectral densities.

## Abstract (web summary)

Multimode optical fibers can be used to observe complex intermodal processes like optical solitons. Here, Eftekhar et al. study accelerated nonlinear interaction in multimode fibers with a tapered core diameter and its effect on the temporal and spectral behavior of the multimode solitons.

## Introduction

Multimode fiber systems offer a promising avenue for overcoming some of the physical limitations associated with single mode structures. The ever-increasing demand for higher data-carrying capacities has incited a flurry of activities^1–4^ that not only led to new classes of active and passive multimode fiber components^5,6^ but it has also prompted a critical rethinking as to how linear/nonlinear processes can be exploited in multimode environments^5–23^—an aspect that has so far remained largely unexplored. In this respect, the added spatial degrees of freedom provide further opportunities at both the scientific and technological level that were previously overlooked and remained unattainable because of technological barriers. From a more fundamental perspective, such hyperdimensional multimode systems provide an ideal testbed for investigating complex linear and nonlinear modal interactions^13–22^—akin to those taking place in many-body molecular dynamics^24^. In recent experiments, nonlinear wave propagation in graded-index multimode fibers (MMFs) was found to lead to a series of unexpected results that are otherwise impossible in single-mode waveguides^19–23,25–27^. These include, for example, the first observation of the so-called geometric parametric instability^22,23,28^, modal condensation as a means to maximize the system’s entropy^23,26,29,30^, formation of multimode soliton “molecules”^19,20^, efficient and instant second-harmonic generation^31^, and visible supercontinuum generation^23,32^ that rivals that obtained from dispersion engineered photonic crystal fibers^33,34^. What makes this possible is the immense spatiotemporal complexity associated with these nonlinear wave-mixing mechanisms in the presence of a large number of modes^20^. Indeed, the prospect of simultaneously locking multiple spatial and longitudinal modes in multimode fiber lasers was successfully demonstrated in a recent work^35^. We would like to emphasize that all the work performed in nonlinear multimode waveguide settings so far, involved fibers with axially uniform cross sections. At this point, little if any is known as to the physics of nonlinear tapered multimode fibers, when operating under either anomalous or normal dispersive conditions.

In this work, we explore accelerated spatiotemporal nonlinear dynamics in long-tapered parabolic-index multimode fibers. As opposed to single-mode fiber tapers, where the axially varying dispersion plays a key role during nonlinear evolution^36,37^, the dispersion in our tapered multimode system remains invariant and the resulting nonlinear interactions are now predominated and drastically altered by the induced accelerated intermodal dynamics—irrespective of the dispersion regime. The ensuing nonlinear modal collisions (via four-wave mixing) in this many-body system leads to a number of interesting results. Under normal dispersive conditions, we observe the formation of upshifted and downshifted gain spectral bands that progressively drift away from the pump wavelength as the intermodal oscillations speed up. In turn, this mechanism leads to the generation of a markedly flat and uniform spectrum that extends over several octaves with large spectral power densities. Meanwhile, in the anomalous dispersion regime, this acceleration process results in a pronounced change of soliton behavior: the emerging multimode solitons (MM-solitons) go through a series of self-adjusting phases that destabilize their spatial distribution along propagation. At the same time, some of these solitons experience an unexpected temporal slowdown in their Raman-induced deceleration, because of a cross-phase modulation (XPM) interaction between the MM-solitons and slow, broadband dispersive waves—a process never observed so far. Finally, we observe a dispersive wave (DW) comb in the spectrum that continuously blue-drifts, not as a result of soliton trapping, but because of a continuously drifting phase-matching condition between DWs and the MM-solitons themselves, that is directly influenced by the accelerated self-imaging behavior. From a fundamental perspective, our results expand our understanding of complex accelerated nonlinear dynamics in tapered graded-index highly-multimode optical waveguides where the constituent modes interact and exchange energy in ways never observed before. In terms of technology, our work may have ramifications in developing tunable fiber sources capable of handling power levels that are orders of magnitude higher than those expected from single-mode arrangements. In the anomalous dispersive region, this tunability can be achieved by exploiting the novel interactions between solitons and DWs. On the other hand, in the normal dispersive regime, the generated supercontinuum in such parabolic multimode tapers not only is relatively uniform, but is also rich in the visible wavelength region. Such high spectral density supercontinuum sources could be ideal for fluorescence microscopy and flow cytometry applications. Even more importantly, the prospect for adjustable high power multimode mode-locked lasers (delivering MWs of peak power) based on taper elements could be another fruitful direction.

## Results

### Spatiotemporal dynamics in anomalously dispersive MMF tapers

In single-mode optical fibers (SMF), temporal solitons represent nonlinear ‘modes’ or stationary states of the underlying (1 + 1)D nonlinear Schrödinger equation—a process enabled by balancing self-phase modulation (SPM) and dispersive effects^38–40^. While in three-dimensional bulk environments, optical solitons happen to be inherently unstable, their stability can be reestablished in multimoded waveguides. Unlike solitons in SMFs that have been intensely investigated over the years, the dynamics of their counterparts in MMFs still poorly understood. Quite recently, multimode solitons have been successfully observed^19^ for the first time in anomalously dispersive parabolic fibers, thus corroborating earlier theoretical results^41^. What makes multimode solitons so complex and rich in dynamics is not only their modal composition but also their intricate intermodal space-time interactions. As opposed to solitons in single mode fiber systems that can be uniquely determined by their energy content, for multimode solitons the situation is considerably more convoluted. This is because the MM-soliton ‘molecules’ can, in principle, take several different forms depending on their modal make-up^20,27^. These composite soliton structures are formed when the constituent modes nonlinearly coalesce after appropriately shifting their frequencies in order to overcome intermodal group-velocity walk-offs. Hence, the soliton fission process, as well as the ensued MM-solitons and corresponding DWs, can be modified by judiciously engineering the modal structure of the input beam^27^. In parabolic-index MMFs, the self-imaging behavior (resulting from the equidistant distribution of propagation eigenvalues) leads to a periodic nonlinear interaction between the soliton modes. This, in turn, gives rise to a broadband sequence of DWs, which in principle can extend into the UV^25^. Here, we investigate how accelerated interactions can affect the soliton fission mechanism and the subsequently generated multimode solitons along with their Cherenkov radiation.

**Fig. 1:**
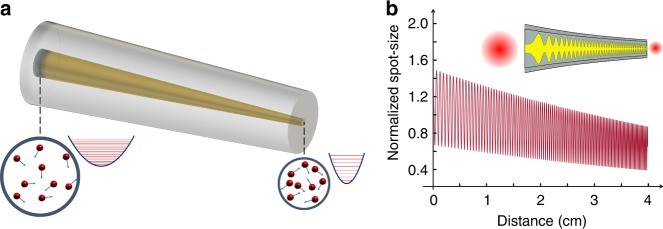
Acceleration of intermodal collisions in a tapered multimode fiber. **a** Schematic of a core-decreasing parabolic multimode fiber. As the fiber core is reduced in size, the spacing between the propagation eigenvalues increases, leading to an acceleration in intermodal collisions and energy exchange. **b** Evolution of the spot-size as a function of the propagation distance in a 4-cm tapered fiber when its core decreases from 40 to 10 µm. In this example, the input spot-size was chosen to be 1.5 times larger than that of the fundamental mode. As shown in **b**, the oscillations in the optical beam diameter experience an acceleration along the propagation direction

To understand these effects, we must first consider the speeding up of the beam expansion/compression cycles in a tapered MMF (Fig. 1a). In our experiments, the core radius, *a*(*z*), of the parabolic MMF was approximately exponentially tapered along the propagation direction, i.e., $a\left( z \right) = a_0\exp ( - \gamma z/2)$, where *a*_0_ is the initial core radius and *γ* is the tapering rate. The core-index potential of this system is described by $n^2 = n_0^2\left( {1 - 2\Delta \left( {r/a_0} \right)^2e^{\gamma z}} \right)$, where Δ represents the relative index change between core and cladding. In seeking Gaussian beam solutions in this parabolic-index arrangement, we use the following ansatz for the spatial part of the optical electric field envelope: $E\left( {r,z} \right) = A\left( z \right)\exp \left[ { - r^2/\left( {2w^2\left( z \right)} \right)} \right]\exp \left\{ {i\left[ {\theta \left( z \right) + r^2F\left( z \right)} \right]} \right\}$ from where one can obtain an Ermakov equation that describes the accelerated evolution of the beam spot-size during propagation in the tapered MMF (Supplementary Note 1). By solving this Ermakov problem, we find:

1 $$\begin{array}{l}w^2 = w_0^2\left[ {AJ_0\left( {\frac{{e^{\beta \xi }}}{\beta }} \right) + BY_0\left( {\frac{{e^{\beta \xi }}}{\beta }} \right)} \right]^2\\ \left[ {1 + \frac{1}{{C^4\beta ^2}}\left( {\tan \left( {\frac{{e^{\beta \xi }}}{\beta } - \phi } \right) - \tan \left( {\frac{1}{\beta } - \phi } \right)} \right)^2} \right]\end{array}$$

In the above expression, *w*_0_ is the input beam waist, $\xi = z\sqrt {2\Delta } /a_0$ represents a normalized propagation distance, and the quantities *β*,*C* and *ϕ* are given by $\beta = \gamma a_0/2\sqrt {2\Delta }$, $C = \sqrt {2\left( {A^2 + B^2} \right)/\pi }$, *ϕ* = π / 4 + tan^−1^(*B/A*), where the coefficients *A* and *B* in these relations are obtained from the input initial conditions. In the case the input beam is incident upon the fiber at its minimum waist, the parameters *A* and *B* can be obtained from $A = - \frac{{\pi w_0}}{{2\beta x_0}}Y_1 \left( {\frac{1}{\beta }} \right),$ $B = \frac{{\pi w_0}}{{2\beta x_0}}J_1\left( {\frac{1}{\beta }} \right)$, where $x_0^2 = \frac{{a_0}}{{\sqrt {2{\mathrm{\Delta }}} k_0n_0}}$, with *x*_0_ being the spot-size of the fundamental mode. In addition, *J*_1_(*x*) and *Y*_1_(*x*) represent first-order Bessel functions of the first and second kind, respectively. Figure 1b depicts the spot-size evolution *w*(*ξ*) as a function of propagation distance. In this example, the input spot-size *w*_0_ was chosen to be 1.5 times larger than that of the fundamental mode *x*_0_. For illustration purposes, we considered a relatively short tapered parabolic-index MMF, where its core radius decreases from 40 to 10 μm within 4 cm. This figure clearly shows that as the fiber narrows down, the beam waist decreases while the intermodal beam oscillation rate experiences an acceleration along the propagation direction. In the presence of nonlinearity, this power density compression implies that the interaction forces among modes are progressively enhanced while experiencing acceleration. As we will see, this acceleration of intermodal oscillations can alter the soliton fission process and drastically change the behavior of the generated DWs and Raman-shifted solitons as well as the soliton modal content.

**Fig. 2:**
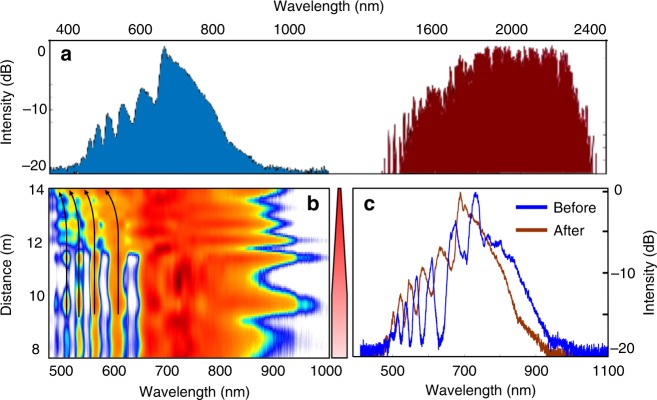
Experimentally observed spectral evolution in a tapered multimode fiber. **a** The spectrum recorded at the output of the tapered multimode fiber when a 100 fs, 1550 nm pulse is used at the input with a peak power of 750 kW. The NIR frequency components are formed because of multimode solitons (red trace) while the visible part arises from dispersive waves (blue trace). **b** Spectral evolution in the visible section as measured using a cutback method. The tapered segment of the multimode fiber is 4 m long (its core radius is reduced from 40 to 10 µm) and is preceded by a 10 m uniform fiber. As the pulses enter the tapered section, dispersive waves experience a continuous blue-drifting of more than 45 nm, indicating a speedup in the intermodal oscillations. The tapered multimode fiber is depicted on the right side, where the darker red color represents an increase in the light intensity in the fiber due to a decrease in core size. **c** The spectrum in **b** is depicted for comparison, before and after the tapered section where blue-shifting of the dispersive wave comb is evident

To experimentally study this tapered MMF system under anomalously dispersive conditions, we use 100 fs pulses (up to 170 nJ in energy) at 1550 nm from an OPO pumped by a mode-locked Ti:Sapphire laser. In these experiments, the input beam waist is 15 µm, thus exciting tens of modes. The parabolic fiber under consideration is comprised of two sections: a 10 m uniform section having a core radius of 40 μm followed by a 4-m tapered segment where the core radius exponentially decreases from 40 to 10 μm. By employing cutback methods, this particular arrangement allows one to monitor and compare the generated nonlinear interactions and spectra in these two different environments. The entire spectrum (at 750 kW) obtained after the taper, is shown in Fig. 2a. The experimentally observed visible side of the spectrum (produced by DWs) is plotted in Fig. 2b. This figure shows, that, in the uniform section, the spectrum finally evolves into a broadband DW comb-like structure, characterized by a series of distinct lines, formed at the soliton fission point. Unlike in single mode fibers where the soliton fission process is accompanied by the generation of a phase-matched narrowband DW line^40,42^, in graded-index MMFs, the resulting DWs are broadband and comb-like, a behavior attributed to the periodic compression/expansion of propagating MM-solitons in graded-index MMFs. As clearly shown in Fig. 2b, the spectrum remains almost invariant as long as the MMF is axially uniform. On the other hand, in the last 4-m tapered stage, as the solitons experience a speedup in their intermodal oscillations, a significant accelerated drift of the DW-combs (black arrows) towards shorter wavelengths is observed. While, DW blue-drifting has been reported in single-mode fibers^43,44^, this was found to result from the trapping of DWs that trail decelerating optical solitons^45^. As a result, this class of DWs eventually shift their central wavelength in order to adjust for now slower propagation velocities—after getting trapped. However, this is not the case in our study since the clusters of DWs are now generated well within the visible portion of the spectrum (500–650 nm), having group velocities that are much slower than those associated with MM-solitons. This rules out any possibility for DW blue-drifting via soliton trapping. Figure 2c compares the output spectra, before and after the tapering section where a blue-drifting of more than 45 nm is observed. The spatial intensity profiles corresponding to the aforementioned combs, before and after the taper section, can be found in Supplementary Note 2.

**Fig. 3:**
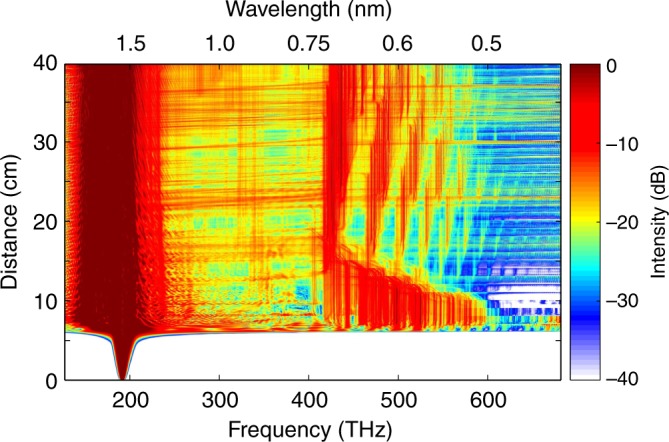
Spectral evolution in a tapered multimode fiber as obtained from a generalized Unidirectional Pulse Propagation Equation simulation. A 400-fs, 1550 nm pulse with a peak power of ~50 kW is injected in a 40 cm long tapered multimode fiber, whose core radius decreases from 40 to 10 µm. After only a few centimeters of propagation, the input pulse undergoes a soliton fission and a sequence of dispersive waves emerges in the visible. Upon further propagation, the generated multimode solitons experience fission-like processes (red horizontal lines) that almost immediately expand the spectrum and generate new blue-drifted dispersive wave lines

To better understand this peculiar DW behavior, we conducted a series of numerical studies and simulations that implicitly take into account modal walk-offs, SPM, cross-phase modulation, four-wave mixing (FWM), Raman and shock effects, etc. Because of the computational complexity of this problem, we model these processes in much shorter taper spans by utilizing generalized Unidirectional Pulse Propagation Equations (gUPPE)^46,47^. The spectrum, as obtained from these simulations, is depicted in Fig. 3. This figure demonstrates that as the soliton breaks up into its multimode soliton constituents, a series of Cherenkov lines (from 500 to 710 nm) is formed as a result of a broadband FWM phase-matching between these multimode solitons and DWs. As the core radius decreases along the fiber-taper, the previously generated solitons are no longer stationary solutions in this progressively changing environment and hence, try to adjust themselves to the local fiber conditions. Our simulations indicate that this instability and self-adjusting mechanism of the emerging higher-order MM-solitons now produces minor fission-like processes through which the solitons shed off some extra energy. This, in turn, leads to the formation of new DW spectral lines, in a way similar to that ensuing close to the primary soliton-fission point. The newly produced DWs are now blue-drifted because of faster intermodal oscillations taking place within multimode solitons in the tapering section (Eq. 1). Since the fiber core radius continuously decreases, the MM-solitons never settle down because of these accelerating dynamics. This causes a cascade of minor fission effects along the fiber taper, leading to a continuous blue-shifting of the emerging DW-combs.

**Fig. 4:**
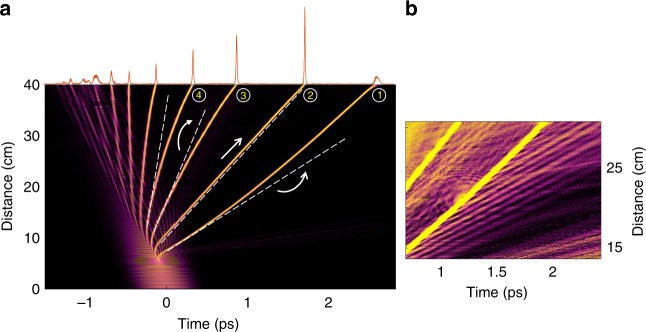
Temporal evolution of a 400-fs pulse at 1550 nm in a tapered multimode fiber as obtained from a generalized Unidirectional Pulse Propagation Equation simulation. **a** Generation and propagation of multimode solitons are displayed in a co-moving temporal window. The parameters used are 400-fs pulse at a wavelength of 1550 nm with a peak power of ~50 kW, injected in a 40 cm long tapered multimode fiber, whose core radius decreases from 40 to 10 µm. The multimode solitons exhibit radically different behaviors as they propagate through the tapered section. The trajectory of the slowest soliton (#1) has a positive curvature, signifying a speed up during propagation. The second soliton (#2) has a zero temporal curvature (and thus no temporal acceleration), while all the subsequent solitons are decelerating. The dashed lines are tangential to the soliton trajectory and represent the instantaneous velocity of the multimode solitons right after soliton fission. **b** A closer look at the trajectory of the first emitted soliton (#1) reveals an emission of dispersive waves as these solitons are continuously perturbed in the tapering section. Because of these dispersive waves, the first soliton experiences a slight bend toward earlier times and hence travels faster

**Fig. 5:**
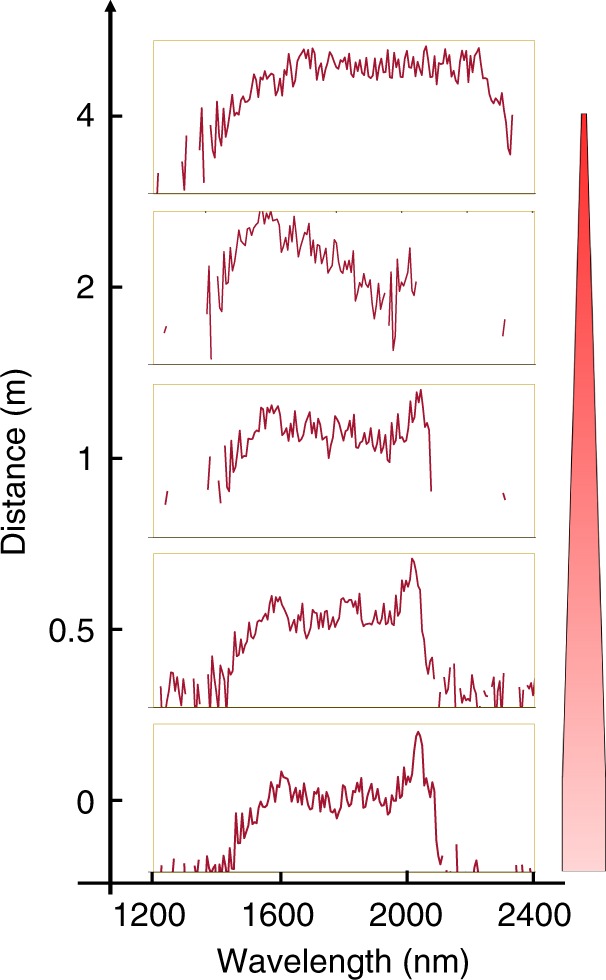
Experimentally observed spectra associated with multimode solitons along the tapered multimode fiber. The NIR spectrum measured along the taper (right) when excited with 100 fs pulses at 1550 nm having a peak power of 750 kW. In the lowest panel, recorded right before the start of the tapered section, the multimode soliton spectrum resides in the long wavelength edge—around 2000 nm. However, after 0.5 m of propagation, the energy is transferred towards shorter wavelengths. After 2 m of propagation, most of the energy is found to be below 1600 nm. Finally, at the end of the taper, the soliton energy is uniformly distributed over a wide spectral window extending from 1500 to 2300 nm. The height of each panel is scaled at 20 dB. The tapered multimode fiber is depicted on the right side, where the darker red color represents an increase in the light intensity in the fiber due to a decrease in core size

We next investigate the impact of these accelerating oscillations on multimode optical solitons. The theoretically anticipated temporal evolution of the input pulse, corresponding to Fig. 3, is depicted in Fig. 4. In this case, after the soliton fission point, the pulse disintegrates into a number of multimode solitons. Interestingly, as the core radius decreases, the generated solitons behave very differently with respect to each other. After a brief period of temporal deceleration, the first emitted soliton starts to eventually accelerate temporally—an indication of soliton blue-shifting. The second soliton travels at a constant velocity and the subsequent solitons experience temporal deceleration. This behavior is unexpected since the Raman induced self-frequency shift (SSFS) tends to slow down solitons by red-shifting their central wavelengths^48^. Temporal re-acceleration of these solitons requires a stronger effect in order to cancel or overcome this Raman-induced temporal deceleration. As previously mentioned, MM-solitons are periodically perturbed in an accelerated fashion (via expansions/compressions) and hence continuously shed Cherenkov DWs as they go through the tapered fiber section. Since these wideband DWs exhibit slower group velocities (as indicated above), they lag behind the generated MM-solitons—thus forcing them to collide with all the previously generated solitons. As shown in recent theoretical studies, the XPM interaction between a soliton and a lagging DW energy packet, can induce a cancelation of SSFS and hence a blue-shifting of the ensued solitons^49,50^. Indeed, a closer look at Fig. 4b reveals that as the first soliton interacts with DWs, it experiences a slight drift toward shorter delay times. This indicates a temporal acceleration of this soliton which is also accompanied by a blue-shifting in the spectral domain. Another interesting aspect associated with this process is a monotonic growth in the average energy residing in the second soliton during propagation. This is in agreement with recent predictions, suggesting that under appropriate conditions a soliton can also absorb energy from DWs and hence grow in intensity^49^. Our experimental observations corroborate this interesting behavior. In this respect, Fig. 5 shows the evolution of the experimentally observed spectrum in the NIR, for five different distances along the MMF taper. While before the tapering section, a significant amount of the total soliton energy happens to reside in the longer wavelength edge (2000 nm) as a result of intrapulse Raman scattering, after 2 m in the taper, a portion of the spectrum shifts towards shorter wavelengths. These results clearly show that the Raman soliton red-shifting has been indeed suppressed through the aforementioned mechanism. This represents the first experimental observation of intrapulse Raman scattering suppression and soliton blue-shifting through a DW interaction—as theoretically predicted in refs. ^49,50^. Figure 5 reveals that, finally, after 3 m of propagation, the spectral energy is uniformly distributed across the NIR window.

As previously reported in several studies, one expects considerable beam self-cleaning in MMFs because of FWM. The spatial beam profile associated with the DW wavelengths, before and after the taper, is relatively speckle-free and Gaussian-like, with most of its energy residing in the fundamental mode (see Supplementary Fig. 1). However, this is not the case for multimode solitons occupying the spectral regions between 1.55–2 µm. The spatial beam profiles corresponding to the various MM-solitons were experimentally characterized at different positions along the MMF taper. While, the soliton beam profiles happen to be quasi-Gaussian in the uniform fiber section, as the solitons enter the tapered section, their modal distribution becomes destabilized and the energy flows towards higher-order modes. This destabilization is attributed to progressively increasing nonlinear effects as a result of tapering, which do not allow the system to attain equilibrium—a condition necessary to observe beam self-cleaning effects. Supplementary Fig. 3 depicts this effect at different positions within the taper. A video displaying this non-equilibrium instability is provided in the Supplementary Movie 1.

**Fig. 6:**
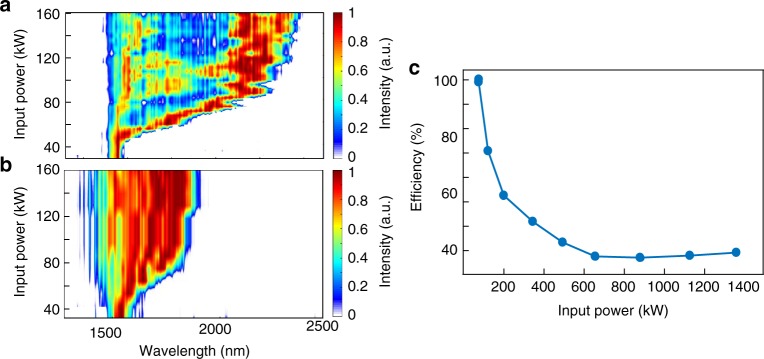
Experimental comparison of soliton red-shifting in a tapered and a uniform multimode fiber. **a** The output spectrum after a 3-m tapered multimode fiber with a core radius decreasing from 30 to 10 µm. As the input power level increases, the soliton experiences a significant red-shifting that extends up to 2300 nm. **b** The recorded output spectrum from a 3-m uniform graded-index multimode fiber having a core radius of 25 µm. In this regime, the solitons experience a subdued red-shifting up to 1900 nm. **c** The transmission efficiency curve as obtained by comparing the fiber output power before and after the tapered segment. As the input power level increases, the efficiency drops. In all cases, 100-fs pulses at 1550 nm are used

The power loss associated with this tapered MMF is measured as a function of the input power and is depicted in Fig. 6c. To estimate the power transmission, the total energy is measured before and after the tapering section. Figure 6c indicates that at relatively low input powers (<70 kW) the transmission through the taper is almost 100%. This high transmission is here attributed to the beam self-cleaning effect that promotes propagation in the lower group of modes, especially in the fundamental mode. However, as the input power is increased to 640 kW, the efficiency significantly drops to ~30%, eventually stabilizing around this value. To understand this behavior, we compared the NIR spectral response of a uniform 3-m long MMF (25 µm core radius) with that of a tapered fiber (3 m in length, and a core radius decreasing from 30 to 10 µm) as a function of input peak power. These results, depicted in Fig. 6a, b, interestingly show that in the tapered case, there is an enhancement in the soliton self-frequency shift, as the input power levels increase. This, in turn, pushes the generated soliton wavelengths into the long-wavelength edge (above 2 µm) where the fiber linear loss happens to be significantly larger. Therefore, the overall loss in the system is expected to increase at higher power levels.

### Spatiotemporal dynamics in normally dispersive MMF tapers

**Fig. 7:**
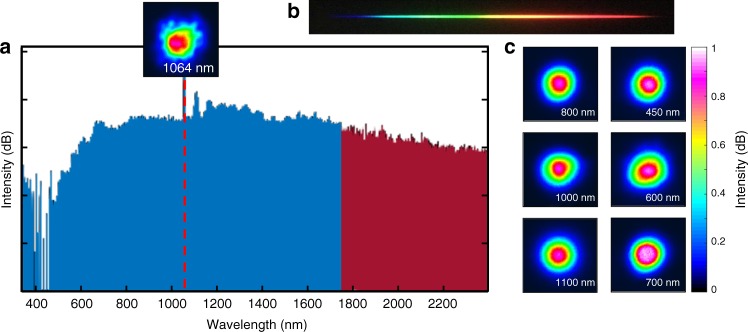
Experimentally obtained supercontinuum spectra from a tapered parabolic-index multimode fiber. **a** The output supercontinuum spectrum after a 15-m long graded-index multimode fiber when its core radius is reduced from 40 to 10 µm. The fiber is excited at 1064 nm in the normal dispersion regime with an input pulse of 400 ps and a peak power of 180 kW. The spectrum happens to be relatively flat, extending from 450 to 2400 nm (more than 2.5 octaves), and was recorded using two different optical spectrum analyzers (blue trace from 350 to 1750 nm, and red trace from 1750 to 2400 nm). Every scale division corresponds to 10 dB. The pump beam profile is shown as an inset. **b** A photograph image of the visible component of the dispersed output spectrum. **c** The beam profiles recorded at different wavelengths of the output supercontinuum. All the beam profiles appear clean and speckle-free, with a significant amount of power residing in the fundamental mode

For this set of experiments, we used in-house fabricated germanium-doped graded-index silica MMFs having different lengths and tapering ratios. In all cases, the fiber index profile remains self-similarly parabolic along the entire length, having a maximum numerical aperture of NA = 0.21. The fibers were excited with a microchip laser pump at 1064 nm, where silica is normally dispersive. This laser delivers 400 ps pulses with a maximum energy of 95 μJ (maximum peak power of 240 kW) at a 500 Hz repetition rate. Laser radiation is coupled into the fibers through a 50 mm focal length lens. The input beam is centered at the fiber front facet, exciting hundreds of modes. Figure 7a shows the resulting supercontinuum spectrum as obtained at the output of a 15-m long MMF taper when its core radius decreases exponentially from 40 to 10 μm. At the pump wavelength, the taper initially supports ~600 modes while at the end of the fiber this number is reduced to ~40. Our experimental results indicate an exceptionally flat and uniform supercontinuum (SC) spectrum, spanning more than 2.5 octaves (from 450 nm to 2400 nm), with a relative variation of less than 10 dB across the entire bandwidth. Such a flat, far-extended spectrum has never been observed before in any other single-mode or multimode fiber setting. To compare the flatness of the spectrum with respect to previous SC observations in similar systems, we introduce a flatness parameter $F = {\int}_{\lambda _1}^{\lambda _2} {d\lambda \left( {S\left( \lambda \right) - \bar S} \right)^2}$, where *S*(*λ*) is the logarithmic SC spectral density as a function of wavelength and $\bar S$ its mean value in the interval [*λ*_1_, *λ*_2_]. In this case, we take *λ*_1_ = 600 nm and *λ*_2_ = 2400 nm. Using this measure, we find that the resulting *F* parameter corresponding to Fig. 7 is ~4.5 times lower than that obtained in ref. ^23^, and ~2 times lower than the one obtained in ref. ^32^, thus indicating a more uniform SC power spectrum. The beam profiles corresponding to different SC wavelengths are also shown in Fig. 7c. In all cases, the experimentally observed beam patterns are quasi-Gaussian and speckle-free. These results suggest that most of the energy at these wavelengths is funneled towards lower order modes.

**Fig. 8:**
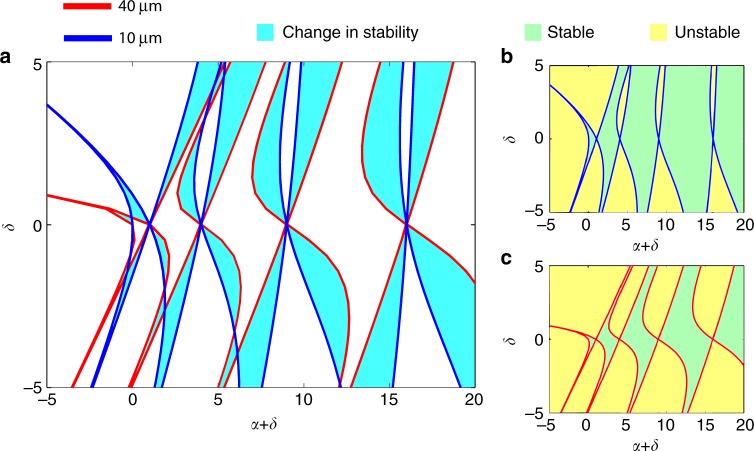
Theoretical stability diagram for a locally uniform multimode fiber. **a** Change in the stability diagram when the fiber radius is reduced from 40 to 10 µm. A reduction in the radius of the multimode fiber shifts the unstable gain regions, thus leading to new spectral lines. **b** and **c** are the stability diagrams corresponding to parabolic multimode fibers with core radii of 40 and 10 µm, respectively. In all cases, the input spot-size was chosen to be 1.5 times that of the fundamental mode and the input peak power was assumed to be 180 kW. The parameters *α* and *δ* are defined as $\alpha = \frac{{a_0^2}}{{2{\mathrm{\Delta }}}}\beta _e^2$, $\delta = \frac{{a_0^2}}{{2{\mathrm{\Delta }}}}\beta _ek_0n_2E_0^2w_0^2$

In order to unravel the origin of this flat supercontinuum generation, it is imperative to first understand how FWM processes or geometric parametric instabilities^22,28,51^ unfold in a uniform graded-index nonlinear MMF. This analysis is pertinent to CW (or in our case broad pulses) excitations in the normal dispersive region. As is well-known, if the input beam is Gaussian, then the CW component undergoes periodic compressions/expansions—resulting from the self-imaging property of parabolic MMFs. This, in turn, has a profound effect on the spectral perturbations *A*(*z*,Ω) located at the sidebands *ω*_0_ ± Ω around the carrier frequency *ω*_0_. Under these conditions, these perturbations obey the following Hill’s equation^51^:

2 $$\frac{{d^2A}}{{d\xi ^2}} + \frac{{a_0^2}}{{2{\mathrm{\Delta }}}}\left[ {\beta _e^2 + \left( {\beta _ek_0n_2E_0^2w_0^2} \right)f\left( \xi \right)} \right]A = 0$$

where *β*_*e*_ = [*β*(*ω*_0_ + Ω)+*β*(*ω*_0_−Ω)]/2−*β*(*ω*_0_) involves all the even terms of the Taylor series expansion of the dispersion profile *β*(*ω*). In the above equation, $\xi = \sqrt {2\Delta } \left( {z/a_0} \right)$ represents a normalized propagation distance, *n*_2_ accounts for the Kerr nonlinear index coefficient, and *k*_0_ = 2π/*λ*_0_, with *λ*_0_ being the pump wavelength. The parameters *E*_0_ and *w*_0_ are fully determined from the initial conditions with the former denoting the initial electric field amplitude and the latter representing the input beam spot-size. The function *f*(*z*)=1/*w*^2^(*z*) corresponds to the beating behavior of light during propagation, where *w*(z) denotes the local beam spot-size. The associated stability diagram (Fig. 8a) and the corresponding gain regions can then be obtained using Floquet theory.

Starting from noise, any frequency component lying in the unstable regions experiences an amplification, leading to a flow of energy from the pump to a particular set of spectral lines. Interestingly, this instability occurs even in the normal dispersion region. The gain experienced by the spectral lines depends on a number of factors including the depth of the nonlinear modulation. Figure 8b, c depict the stability diagrams corresponding to the entry and exit regions of a tapered MMF. The parameters used to obtain these diagrams are identical to those involved in our experiment at a peak power level of 180 kW. As it is evident from Fig. 8, as the core radius decreases from 40 to 10 µm, the unstable (gain) frequency regions start to drift in a substantial way, thus sweeping the entire spectrum. This explains the flat supercontinuum generation observed in our experiment. In essence, this temporal accelerated drift of the unstable regions allows the FWM to equally populate the SC.

**Fig. 9:**
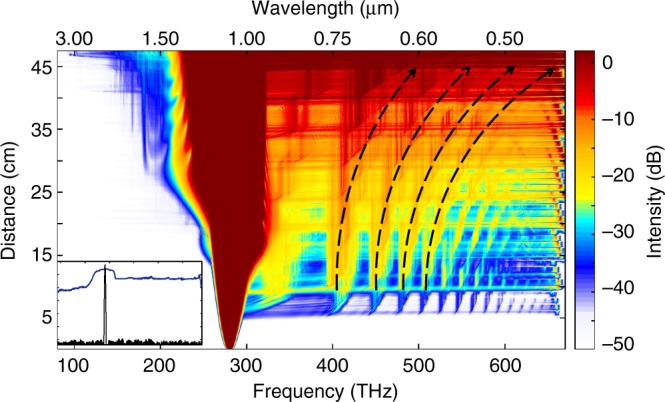
Generalized Unidirectional Pulse Propagation Equation simulation of the spectral evolution in a tapered multimode fiber when excited in the normal dispersion regime. A 0.5 m long multimode fiber taper is excited with a 300 kW, 400 fs pulse, at 1064 nm. After only a few centimeters of propagation (~ 5 cm), a series of sidebands emerge. As the input pulse traverses the tapering section, an acceleration of intermodal collisions occurs. As a result, the newly generated sidebands drift away from the pump (dashed arrows), thus sweeping the entire spectrum. This leads to a flat and uniform supercontinuum. The inset compares the spectrum of the input pulse (black) with the output supercontinuum (blue)

To simulate these effects, we use gUPPE methods. The parameters employed in our simulations correspond to those in our experiment, only this time the length of the taper is assumed to be 0.5 m (to reduce computational complexity). The evolution of the spectrum during propagation is depicted in Fig. 9. After a few centimeters of propagation, a series of sidebands emerge – as expected from geometric parametric instability. For longer propagation distances, where the beam oscillations speed up, all the generated sidebands start to drift away from the pump in an accelerated fashion (dashed arrows in Fig. 9). These accelerated sidebands subsequently merge and form a uniform spectrum. The inset in Fig. 9 shows the spectrum at the input and the output of the fiber, as obtained from gUPPE. In addition, as the spectrum extends into the anomalous dispersion regime, formation of solitons is imminent. This process leads to a relatively uniform spectrum in the NIR—as it was previously shown in Fig. 5. This can explain the extent of the spectrum in the NIR (Fig. 7a). Note, that this sideband generation occurs despite the fact that the modal oscillations are no longer periodic. Nevertheless, this instability can only be encouraged as long as the tapering takes place in an adiabatic manner—thus allowing the sidebands to efficiently grow. The impact of the adiabaticity on the spectral evolution is provided in Supplementary Note 4.

In conclusion, we have investigated accelerated nonlinear intermodal interactions in core decreasing multimode fibers. We have demonstrated that this spatiotemporal acceleration can have a significant effect on the temporal and spectral behavior in both dispersion regimes. In the anomalous dispersive region, this acceleration leads to blue-drifting dispersive wave combs, while under normal dispersive conditions we observe a generation of accelerating frequency bands that dynamically and uniformly sweep the entire spectrum. This, in turn, can be utilized to produce a notably flat and uniform supercontinuum, extending over 2.5 octaves. Our results could pave the way towards a new class of optical sources based on the aforementioned nonlinear acceleration dynamics.

## Discussion

Historically, the field of nonlinear fiber optics has revolved mainly around single-mode optical fibers. Taking a fresh look at multimode fibers could provide new opportunities at both the scientific and technological levels. The additional spatial degrees of freedom offered by MMFs can indeed lead to a host of new phenomena. These include, for example, the emergence of geometric parametric instabilities, MM-soliton formation, and beam self-cleaning. As indicated in our study, judiciously designed tapered multimode fibers can lead to accelerated nonlinear interactions, capable of generating new spectral lines, anywhere between the deep blue and IR wavelengths. In this respect, a gradual acceleration of these intermodal oscillations can be employed to induce an ultrabroad and uniform supercontinuum. In principle, because of larger core sizes, the SC produced in MMFs can have orders of magnitude higher spectral densities—as required in many applications. This same scheme can be used in fibers based on other material systems, such as chalcogenides that are considerably more transparent in IR. In the anomalous dispersive region, DW generation from accelerated soliton oscillations can lead to a significant blue-shift. This mechanism could be exploited in hollow-core photonic crystal fiber structures having minimal loss in the blue and near UV regions.

Another fundamental aspect considered in our study is associated with the complex dynamics of multimode spatiotemporal solitons in multimode fiber tapers. Interestingly, the nonlinear interaction between multimode solitons and slow propagating dispersive waves can mitigate or reverse the Raman-induced soliton self-frequency shift and the subsequent deceleration of solitons. In this same tapered environments, the spatially clean multimode solitons were found to destabilize, thus promoting energy transfer to higher-order modes.

Our observations indicate that the complexity of accelerated nonlinear dynamics in multimode fiber tapers can be potentially exploited for a number of applications, ranging from high power spatiotemporal mode-locked multimode fiber lasers to tunable sources with high spectral densities and tailored wavelength characteristics. Finally, our work provides valuable insight into the physics and complexity of nonlinear wave propagation in axially non-uniform multimode environments.

## Methods

### Simulations

The simulations were conducted using generalized unidirectional pulse propagation equations (gUPPE). The primary fiber parameters used in the simulations were the same (except for the length) as the tapered parabolic-index MMFs used in our experiments. In this respect, the relative index change was assumed to be Δ ≈ 1.6 × 10^−3^ and the silica Kerr coefficient was taken to be *n*_2_ = 2.9 × 10^−20^ m^2^W^−1^. In all cases, the core radius was reduced from 40 to 10 µm, as in the experiments. In addition, all nonlinear and linear processes such as modal walk-offs, self-focusing, self-phase modulation, cross-phase modulation, FWM, Raman, and shock effects were accounted for in the simulations. The input pulse in the anomalous regime was centered around 1550 nm (0.193 PHz) and had a peak power of 500 kW. On the other hand, in the case of normal dispersion, the pulses were centered around 1064 nm (0.282 PHz), having a peak power of ~300 kW. In all our simulation, the pulse-widths (Figs. 3, 4 and 9) were assumed to be 400 fs. The longitudinal step size was set at Δz ≈ 1 μm. For undertaking such computationally demanding simulations, we used parallel computing, provided by the XSEDE supercomputer facility^52^.

### Experiments

The tapers used in both experiments utilized in-house fabricated germanium-doped graded-index silica multimode optical fibers having a range of fiber lengths and tapering ratios. The pulses were coupled to the fiber with up to 140 nJ (1.4 MW peak power) from a Ti:Sapphire laser source (1.55 µm) and with up to 72 µJ (180 kW peak power) form a microchip laser pump (1.064 µm). A three-axis translation stage and a 5 cm focal length lens were used to couple laser beams into the fibers. In all cases, the fibers had a NA = 0.21. The ensuing spectrum was measured with visible and IR spectrometers. Beam profiles at the output of the fibers were captured using visible and IR cameras. Several spectral filters were used to obtain the spatial distribution of the output beams at different wavelengths. For the cutback experiment, all measurements were recorded under the same initial conditions.

## Supplementary information

Supplementary Information

Description of Additional Supplementary Files

Supplementary Movie 1

## Data Availability

The data that support the plots within this paper and other findings of this study are available from the corresponding author upon reasonable request.

## Code availability

The simulation code, developed in the frame of refs. ^46,47^, is available upon reasonable request.
