# Supplementary material for: Accelerated nonlinear interactions in graded-index multimode fibers
Source: Nat Commun. 2019 Apr 9;10:1638. doi: 10.1038/s41467-019-09687-9 (PMC6456603; doi:10.1038/s41467-019-09687-9)
Supplement: Supplementary file 1 — Supplementary Information [file 41467_2019_9687_MOESM1_ESM.pdf]

Supplementary Information

Accelerated nonlinear interactions in graded-index multimode fibers

Eftekhar et. al.

Supplementary Information

Accelerated nonlinear interactions in graded-index multimode fibers

M. A. Eftekhar, Z. Sanjabi-Eznaveh, H. Lopez-Aviles, S. Benis, J. E. Lopez, M. Kolesik, F. Wise, R. Amezcua-Correa, D. N. Christodoulides

Supplementary Note 1 | Calculating the behavior of a beam waist in a longitudinally engineered multimode fiber.

The refractive index profile of a non-uniform optical fiber can be represented as below:

$$n = n_0\left(1 - \Delta\left(\frac{r}{a}\right)^2 f(z)\right) \tag{1}$$

By replacing the above index profile in the Helmholtz equation ($\nabla^2 E + k_0^2 n^2 E = 0$) we acquire the field evolution equation in a fiber profile with z-dependent core radii. Next, we use the slowly varying envelope approximation (SVEA) $E(x,y,z) = \Phi(x,y,z)e^{ik_0 n_0 z}$. By decomposing the operator ∇^2 to longitudinal and transverse components ($\nabla^2 = \partial_{zz} + \nabla_\perp^2$) we have:

$$\nabla^2 E + k_0^2 n^2 E = (\partial_{zz} + \nabla_\perp^2)E + k_0^2 n^2 E \tag{2}$$

$$\Phi_{zz} + 2ik_0 n_0 \Phi_z - k_0^2 n_0^2 \Phi + \nabla_\perp^2 \Phi + k_0^2 n^2 \Phi = 0 \tag{3}$$

By replacing the index profile $n(z)$ we get:

$$\Phi_{zz} + 2ik_0 n_0 \Phi_z - k_0^2 n_0^2 \Phi + \nabla_\perp^2 \Phi + k_0^2 n_0^2 \Phi - 2\Delta\left(\frac{r}{a}\right)^2 f(z) k_0^2 n_0^2 \Phi = 0 \tag{4}$$

$$i\Phi_z + \frac{1}{2\mathrm{k}_0 n_0}\nabla_\perp^2 \Phi - \Delta\left(\frac{r}{a}\right)^2 f(z) k_0 n_0 \Phi = 0 \tag{5}$$

One way to solve this differential equation is through separation of variables:

$$\Phi = F(x,z)G(y,z) \tag{6}$$

Which gives

$$\frac{iF_z}{F} + \frac{iG_z}{\mathrm{G}} + \frac{1/\mathrm{F}}{2\mathrm{k}_0 n_0}\nabla_\perp^2\mathrm{F} + \frac{1/\mathrm{G}}{2\mathrm{k}_0 n_0}\nabla_\perp^2\mathrm{G} - \Delta\left[\left(\frac{x}{a}\right)^2 + \left(\frac{y}{a}\right)^2\right] f(z)k_0 n_0 = 0 \qquad (7)$$

Separating the terms independent of *x* and *y* gives:

$$iF_z + \frac{1}{2\mathrm{k}_0 n_0}F_{xx} - \Delta\left(\frac{x}{a}\right)^2 f(z)k_0 n_0 F = 0 \qquad (8)$$

$$iG_z + \frac{1}{2\mathrm{k}_0 n_0}G_{yy} - \Delta\left(\frac{y}{a}\right)^2 f(z)k_0 n_0 G = 0 \qquad (9)$$

Since both equations are essentially identical, one needs to solve only one of them. The evolution equation under consideration is as below:

$$iu_z + \frac{1}{2\mathrm{k}_0 n_0}u_{xx} - \Delta\left(\frac{x}{a}\right)^2 f(z)k_0 n_0 u = 0 \qquad (10)$$

The above equation is normalized by applying the following normalization parameters.

$$\xi = \frac{z}{a}\sqrt{2\Delta}, \qquad s = \frac{x}{x_0}, \qquad x_0^2 = \frac{a}{\sqrt{2\Delta}}\frac{1}{k_0 n_0} \qquad (11)$$

where x_0 is the spot-size of the fundamental mode. After applying the normalization parameters one gets:

$$i\frac{\partial u}{\partial \xi} + \frac{1}{2}\frac{\partial^2 u}{\partial s^2} - \frac{s^2}{2}f(\xi)u = 0 \qquad (12)$$

We assume a Gaussian-type solution for the evolution equation as below

$$u(s,\xi) = A(\xi)\ e^{-\frac{s^2}{2w^2(\xi)}}\ e^{i[\theta(\xi)+s^2F(\xi)]} \qquad (13)$$

where $w(\xi)$ is the beam waist.

$$u_\xi = \left[A' + A\left(i\theta' + is^2F' + \frac{s^2}{w^3}w'\right)\right] e^{-\frac{s^2}{2w^2(\xi)}}\ e^{i[\theta(\xi)+s^2F(\xi)]} \qquad (14)$$

$$u_{ss} = A\left(\frac{S^2}{w^4} - \frac{i4s^2F}{w^2} - 4s^2F^2 + 2iF - \frac{1}{w^2}\right)e^{-\frac{s^2}{2w^2(\xi)}}\ e^{i[\theta(\xi)+s^2F(\xi)]} \qquad (15)$$

$$iA' - A\theta' - As^2F' + i\frac{s^2}{w^3}Aw' + \frac{As^2}{2w^4} - \frac{i2s^2FA}{w^2} - 2s^2F^2A + iFA - \frac{A}{2w^2} - \frac{s^2}{2}f(\xi)A = 0 \qquad (16)$$

By separating real and imaginary parts as well as terms with different powers of s, we have

$$2F' = \frac{1}{w^4} - 4F^2 - f(\xi) \tag{17}$$

$$\theta' + \frac{1}{2w^2} = 0 \tag{18}$$

$$\frac{w'}{w^3} - \frac{2F}{w^2} = 0 \tag{19}$$

$$A' + FA = 0 \tag{20}$$

From Eq. (19):

$$w' - 2Fw = 0 \tag{21}$$

$$F = \frac{w'}{2w} \tag{22}$$

Replacing F in Eq. (20) gives:

$$A' + \frac{w'}{2wA} = 0 \tag{23}$$

$$2A'Aw + w'A^2 = 0 \tag{24}$$

$$(A^2w)' = 0 \tag{25}$$

$$A^2w = const. \tag{26}$$

Equation (26) is a manifestation of conservation of energy. By replacing F and F' in Eq. (17):

$$4F^2 = \frac{w'^2}{w^2} \tag{27}$$

$$2F' = \frac{w''}{w} - \frac{w'^2}{w^2} \tag{28}$$

$$2F' = \frac{1}{w^4} - 4F^2 - f(\xi) \tag{29}$$

$$\frac{w''}{w} - \frac{w'^2}{w^2} = \frac{1}{w^4} - \frac{w'^2}{w^2} - f(\xi) \tag{30}$$

$$w^{''} + f(\xi)w = \frac{1}{w^3} \tag{31}$$

$$\frac{d^2w(\xi)}{d\xi^2} + f(\xi)w(\xi) = \frac{1}{w(\xi)^3} \tag{32}$$

Equation (32) is called Ermakov (Yermakov) equation. To solve the Ermakov equation, one needs to first solve the homogenous second order linear equation:

$$y_{xx}^{''} + f(x)y = 0 \tag{33}$$

Solving this equation gives Q as the solution,

$$Q_{xx}^{''} + f(x)Q(x) = 0 \tag{34}$$

Then applying the transformation below simplifies the equation,

$$\zeta = \int \frac{dx}{Q^2(x)}, \qquad z = \frac{y}{Q(x)} \tag{35}$$

which gives

$$z_{\zeta\zeta}^{\prime\prime} = az^{-3} \tag{36}$$

The exact solution to the equation above is given by

$$C_1 y^2 = aQ^2 + Q^2\left(C_2 + C_1 \int \frac{dx}{Q^2(x)}\right)^2 \tag{37}$$

To solve the above equation we need to define the function $f(\xi)$ first. From Eq. (1), we know that:

$$n^2 = n_0^2\left(1 - 2\Delta\left(\frac{r}{a_0}\right)^2 f(z)\right) \tag{38}$$

$$a(z) = a_0 e^{-\alpha z} \tag{39}$$

$$f(z) = e^{2\alpha z} \tag{40}$$

For example, if the fiber is gradually changing in radius from 30 μm to 10 μm over 25 m, then $\alpha(0) = a_0 = 30$ μm, $a(25) = a_0 e^{-25\alpha} = 10$ μm, $e^{-25\alpha} = 1/3$, $\alpha = \ln(3)/25 = 0.0439445$. From the normalization parameters, we have $z = \xi a_0/\sqrt{2\Delta}$

$$f(\xi) = e^{2\alpha\frac{a_0}{\sqrt{2\Delta}}\xi} \tag{41}$$

To see the evolution of the beam waist along propagation, the following equation must be first solved:

$$\frac{d^2w(\xi)}{d\xi^2} + e^{2\alpha\frac{a_0}{\sqrt{2\Delta}}\xi}w(\xi) = \frac{1}{w(\xi)^3} \tag{42}$$

For simplicity, we apply the change of variable $\beta = \alpha\frac{a_0}{\sqrt{2\Delta}}$.

The homogeneous equation is as below:

$$\frac{d^2Q(\xi)}{d\xi^2} + e^{2\beta\xi}Q(\xi) = 0 \tag{43}$$

We know that for the following equation

$$y''_{xx} + ae^{\lambda x}y = 0, \quad \lambda \neq 0 \tag{44}$$

The solution is

$$y = C_1J_0(z) + C_2Y_0(z), \quad z = \frac{2}{\lambda}\sqrt{a}e^{\lambda x/2} \tag{45}$$

Thus, the solution to this equation will be

$$Q(\xi) = AJ_0\left(\frac{e^{\beta\xi}}{\beta}\right) + BY_0\left(\frac{e^{\beta\xi}}{\beta}\right) \tag{46}$$

Having $Q(\xi)$ we know that the complete solution will be

$$C_1w^2 = Q^2 + Q^2\left(C_2 + C_1\int\frac{d\xi}{Q^2(\xi)}\right)^2 \tag{47}$$

Taking the integral of the inverse square of $Q(\xi)$ is very difficult nay impossible. The question is if there is any other way to simplify this integral. We should first check the typical values for β. For our current fiber $\beta = 0.0439445 \times 30 \times 10^{-6}/\sqrt{2 \times 0.010174} = 9.242 \times 10^{-6}$, and $1/\beta = 1.08 \times 10^5$.

Given that Bessel arguments are scaled with $1/\beta$ implies that we can use asymptotic forms:

$$J_\nu(z) \sim \left(\frac{2}{\pi z}\right)^{1/2}\left[\cos\left(z - \frac{1}{2}\pi\nu - \frac{1}{4}\pi\right)\sum_{m=0}^{\infty}\frac{(-1)^m(\nu,2m)}{(2m)^{2m}} - \sin\left(z - \frac{1}{2}\pi\nu - \frac{1}{4}\pi\right)\sum_{m=0}^{\infty}\frac{(-1)^m(\nu,2m+1)}{(2z)^{2m+1}}\right] \tag{48}$$

$$Y_\nu(z) \sim \left(\frac{2}{\pi z}\right)^{1/2} \left[\sin\left(z - \frac{1}{2}\pi\nu - \frac{1}{4}\pi\right) \sum_{m=0}^{\infty} \frac{(-1)^m(\nu, 2m)}{(2m)^{2m}} + \cos\left(z - \frac{1}{2}\pi\nu - \frac{1}{4}\pi\right) \sum_{m=0}^{\infty} \frac{(-1)^m(\nu, 2m+1)}{(2z)^{2m+1}}\right] \quad (49)$$

where

$$(\nu, m) = \frac{\Gamma\left(\nu + m + \frac{1}{2}\right)}{m!\,\Gamma\left(\nu - m + \frac{1}{2}\right)} \quad (50)$$

Since we are dealing with J_0 and Y_0 so $\nu = 0$. In this case $\sum_{m=0}^{\infty} \frac{(-1)^m(\nu,2m)}{(2m)^{2m}}$ is 1 for $m = 0$ and 0 for $m > 1$, and $\sum_{m=0}^{\infty} \frac{(-1)^m(\nu,2m+1)}{(2z)^{2m+1}}$ is 0 for all m as long as $\nu = 0$.

So we are going to have

$$J_\nu(z) \sim \left(\frac{2}{\pi z}\right)^{1/2} \cos\left(z - \frac{1}{4}\pi\right) \quad (51)$$

$$Y_\nu(z) \sim \left(\frac{2}{\pi z}\right)^{1/2} \sin\left(z - \frac{1}{4}\pi\right) \quad (52)$$

And asymptotically, we get the following equation:

$$Q(\zeta) = \left(\frac{2}{\pi\zeta}\right)^{1/2} \left[A\cos\left(\zeta - \frac{1}{4}\pi\right) + B\sin\left(\zeta - \frac{1}{4}\pi\right)\right] \quad (53)$$

We can further simplify the above equations

$$Q(\zeta) = \left(\frac{2}{\pi\zeta}\right)^{1/2} \left[A\cos\left(\zeta - \frac{1}{4}\pi\right) + B\sin\left(\zeta - \frac{1}{4}\pi\right)\right] \quad (54)$$

$$Q(\zeta) = \left(\frac{2}{\pi\zeta}\right)^{1/2} \sqrt{A^2 + B^2} \left[\frac{A}{\sqrt{A^2 + B^2}}\cos\left(\zeta - \frac{1}{4}\pi\right) + \frac{B}{\sqrt{A^2 + B^2}}\sin\left(\zeta - \frac{1}{4}\pi\right)\right] \quad (55)$$

Given that $\cos(\alpha - \beta) = \cos(\alpha)\cos(\beta) + \sin(\alpha)\sin(\beta)$, where $\cos(\beta) = \frac{A}{\sqrt{A^2+B^2}}$, $\sin(\beta) = \frac{B}{\sqrt{A^2+B^2}}$, $\tan(\beta) = \frac{B}{A}$, $\beta = \mathrm{atan}\left(\frac{B}{A}\right)$, gives

$$Q(\zeta) = \frac{C_Q}{\sqrt{\zeta}}[\cos(\zeta - \phi)] \quad (56)$$

where $C_Q = \sqrt{\frac{2}{\pi}}\sqrt{A^2 + B^2}$, $\phi = \frac{\pi}{4} + \text{atan}\left(\frac{B}{A}\right)$ and $\zeta = \frac{e^{\beta\xi}}{\beta}$.

Now we proceed to solve the integral $\int \frac{d\xi}{Q^2(\xi)}$, where $d\xi = \frac{d\zeta}{\beta\zeta}$,

$$\int \frac{d\zeta}{Q^2(\zeta)} = \int \frac{\zeta \frac{d\zeta}{\beta\zeta}}{C_Q^2 \cos^2(\zeta - \phi)} = \frac{1}{C_Q^2\beta} \int \frac{d\zeta}{\cos^2(\zeta - \phi)} = \frac{1}{C_Q^2\beta} \tan(\zeta - \phi) + const \qquad (57)$$

By replacing ζ with ξ we get

$$\int \frac{d\xi}{Q^2(\xi)} = \frac{1}{C_Q^2\beta} \tan\left(\frac{e^{\beta\xi}}{\beta} - \phi\right) + const \qquad (58)$$

We are now left with computing the coefficients from the initial conditions. We consider a special case of $C_2 = 0$

$$C_1 w^2 = Q^2 \left(1 + C_1^2 \left[\int_0^\xi \frac{d\xi}{Q^2(\xi)}\right]^2\right) \qquad (59)$$

at $z = 0$ ($\xi = 0$), $w(0) = w_0$

$$C_1 w^2 = Q^2 \qquad (60)$$

$$Q(\xi) = AJ_0\left(\frac{e^{\beta\xi}}{\beta}\right) + BY_0\left(\frac{e^{\beta\xi}}{\beta}\right) \qquad (61)$$

$$C_1 = \frac{\left[AJ_0\left(\frac{1}{\beta}\right) + BY_0\left(\frac{1}{\beta}\right)\right]^2}{w_0^2} \qquad (62)$$

Our second assumption is that the incoming wave is incident on the fiber at its minimum waist $w' = 0$.

$$2C_1 w\, w' = 2Q\, Q' \left(1 + C_1^2 \left[\int_0^\xi \frac{d\xi}{Q^2(\xi)}\right]^2\right) + 2Q^2 C_1^2 \left[\int_0^\xi \frac{d\xi}{Q^2(\xi)}\right] \frac{1}{Q^2} \qquad (63)$$

At $\xi = 0$, $\int_0^\xi \frac{d\xi}{Q^2(\xi)} = 0$ so:

$$2C_1 w\, w' = 2Q\, Q' \qquad (64)$$

So $Q' = 0$,

$$\frac{dQ(\xi)}{d\xi} = e^{\beta\xi}\left[AJ_0'\left(\frac{e^{\beta\xi}}{\beta}\right) + BY_0'\left(\frac{e^{\beta\xi}}{\beta}\right)\right] \qquad (65)$$

Evaluating the above expression at $\xi = 0$ gives

$$\frac{dQ(\xi)}{d\xi}|_{\xi=0} = AJ_0'\left(\frac{1}{\beta}\right) + BY_0'\left(\frac{1}{\beta}\right) = 0 \qquad (66)$$

We can always normalize the coefficients and consider $C_1 = 1$, then $Q(0) = w_0$, which gives

$$AJ_0\left(\frac{1}{\beta}\right) + BY_0\left(\frac{1}{\beta}\right) = w_0 \qquad (67)$$

$$AJ_0'\left(\frac{1}{\beta}\right) + BY_0'\left(\frac{1}{\beta}\right) = 0 \qquad (68)$$

Given the above equations *A* and *B* are easily calculated as below:

$$A = \frac{w_0 Y_0'\left(\frac{1}{\beta}\right)}{J_0\left(\frac{1}{\beta}\right)Y_0'\left(\frac{1}{\beta}\right) - J_0'\left(\frac{1}{\beta}\right)Y_0\left(\frac{1}{\beta}\right)} \qquad (69)$$

$$B = \frac{-w_0 J_0'\left(\frac{1}{\beta}\right)}{J_0\left(\frac{1}{\beta}\right)Y_0'\left(\frac{1}{\beta}\right) - J_0'\left(\frac{1}{\beta}\right)Y_0\left(\frac{1}{\beta}\right)} \qquad (70)$$

According to Wronskian relation

$$J_\alpha(x)Y_\alpha'(x) - J'_\alpha(x)Y_\alpha(x) = \frac{2}{\pi x} \qquad (71)$$

then in our case we simply have:

$$J_0\left(\frac{1}{\beta}\right)Y_0'\left(\frac{1}{\beta}\right) - J_0'\left(\frac{1}{\beta}\right)Y_0\left(\frac{1}{\beta}\right) = \frac{2\beta}{\pi} \qquad (72)$$

finally, the coefficients are found as below

$$A = \frac{\pi w_0}{2\beta}Y_0'\left(\frac{1}{\beta}\right) = -\frac{\pi w_0}{2\beta}Y_1\left(\frac{1}{\beta}\right) \qquad (73)$$

$$B = -\frac{\pi w_0}{2\beta}J_0'\left(\frac{1}{\beta}\right) = +\frac{\pi w_0}{2\beta}J_1\left(\frac{1}{\beta}\right) \qquad (74)$$

Supplementary Note 2 | Output beam profiles corresponding to the accelerated dispersive waves.

Supplementary Figure 1 shows the beam profiles of four different wavelengths before and after the tapered section. The beam profiles before the tapering section are clean and Gaussian-like. This can be a result of the spatial beam self-cleaning happening in MMFs. The beam profiles of the blue-drifted DWs are depicted in Supplementary Figure 1 (e-h). The beam distribution stays clean and mostly confined in the fundamental mode even after going through an acceleration of oscillations.

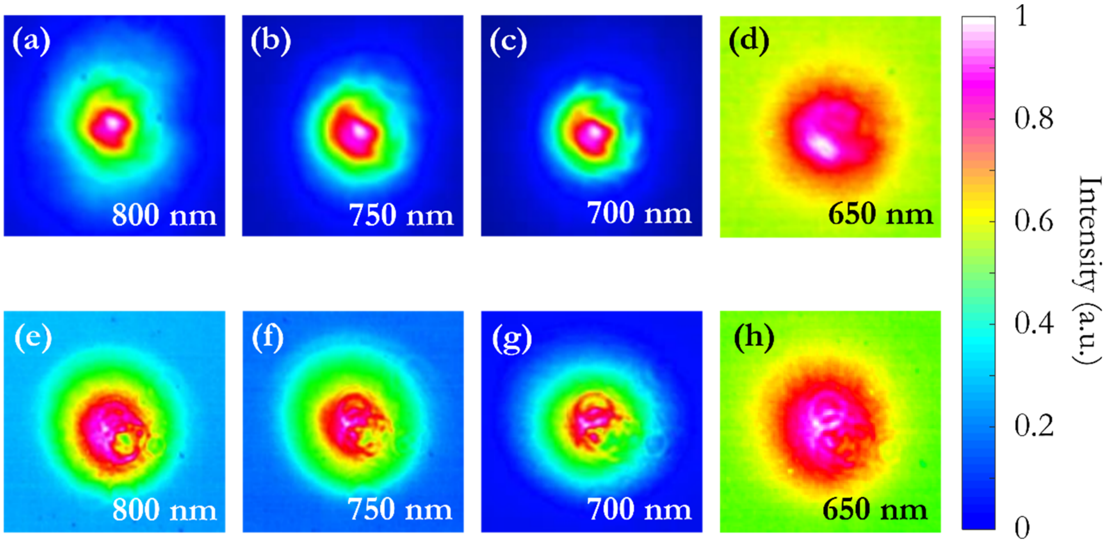

Supplementary Figure 1 | Beam profiles corresponding to dispersive waves before and after the tapered section. a-d Beam profiles at 4 different wavelengths 800, 750, 700 and 650 nm before the tapered section and after 10 m of propagation through the uniform fiber. The beam profiles are clean with most of energy in the fundamental mode. **e-h** Beam profiles after the tapering section at the same four wavelengths. The dispersive waves generated as a result of acceleration of solitons are clean and mostly residing in the fundamental mode.

Supplementary Note 3 | Output beam profiles corresponding to the Raman shifted solitons.

The beam profile distribution for Raman shifted solitons for three sample wavelengths – recorded in the output of the 10-m uniform graded-index MMF – are shown in Supplementary Figure 2. All the profile distributions at all wavelengths were clean and stable. However, as soon as the solitons enter the tapered section and experience an acceleration in the intermodal interaction of their constituent modes, they lose their stability. This unstable behavior is demonstrated in 8 frames in Supplementary Figure 3. For this measurement, the output spectrum was filtered at 1600 nm. As it is evident in this figure, the beam is no longer Gaussian-like. In other words, the soliton energy that used to stay in the fundamental mode continuously jumps to different modes and populates the higher-order modes.

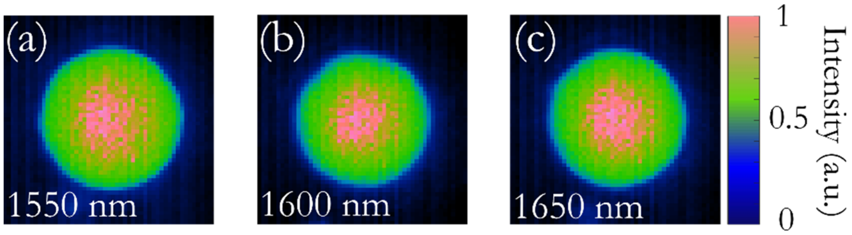

Supplementary Figure 2 | Beam profile of the Raman-shifted solitons at three sample wavelengths. **a-c** Beam profiles captured right before the tapered section. In all cases, the beam distributions are Gaussian-like, clean and stable.

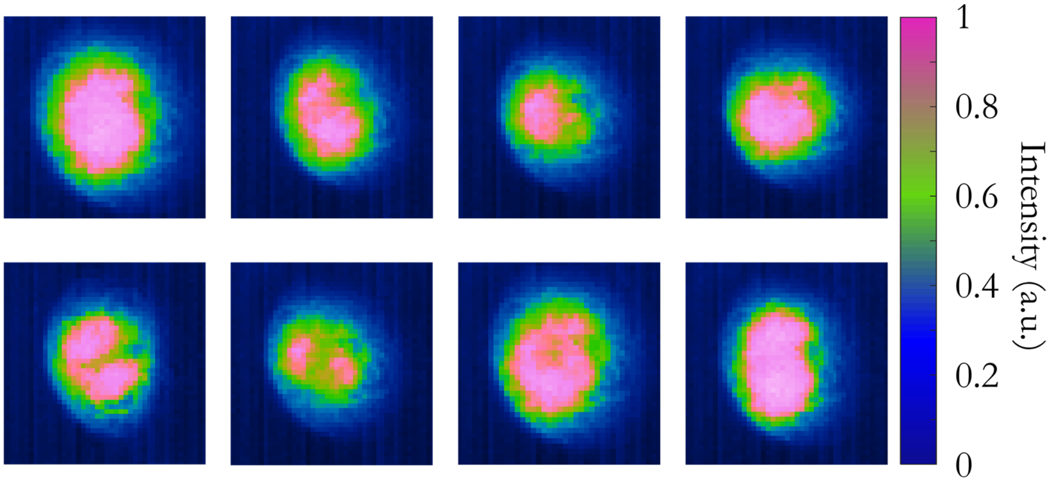

Supplementary Figure 3 | Beam profiles of the Raman-shifted solitons measured after the tapering section. These beam profiles are measured at a sample distance of 2 m after the onset of the tapered section. The beam profiles are recorded at 1600 nm for 8 different instants. The beam is very unstable and the energy continuously jumps among different modes. A ring pattern and the beam profile of LP_{11} mode are evident in this figure.

Supplementary Note 4 | The effect of taper length on the generated spectrum.

The periodic compression/expansion of a propagating beam in a parabolic-index multimode fiber leads to a sideband generation process. The efficiency of this process depends upon many factors including the perfect periodic oscillations. As the oscillation deviate from a perfect periodicity, efficiency drops. Tapering rate determines the efficiency of the process. In Supplementary Figure 4, a 1-m long tapered fiber with a core radius changing rapidly from 25 to 10 μm has been illuminated with different power levels of input beam. In all cases, the sideband generation is suppressed due to a fast tapering rate. Supplementary Figures 5 and 6, show the output spectra of two fiber tapers of 3 and 5 m. These results show that as the tapering rate gets smaller and the taper length increases, the output spectrum will be more flat and uniform. Also, to push the short wavelength edge of the spectrum towards blue and UV, one needs to use a tapered fiber with smaller terminal radius.

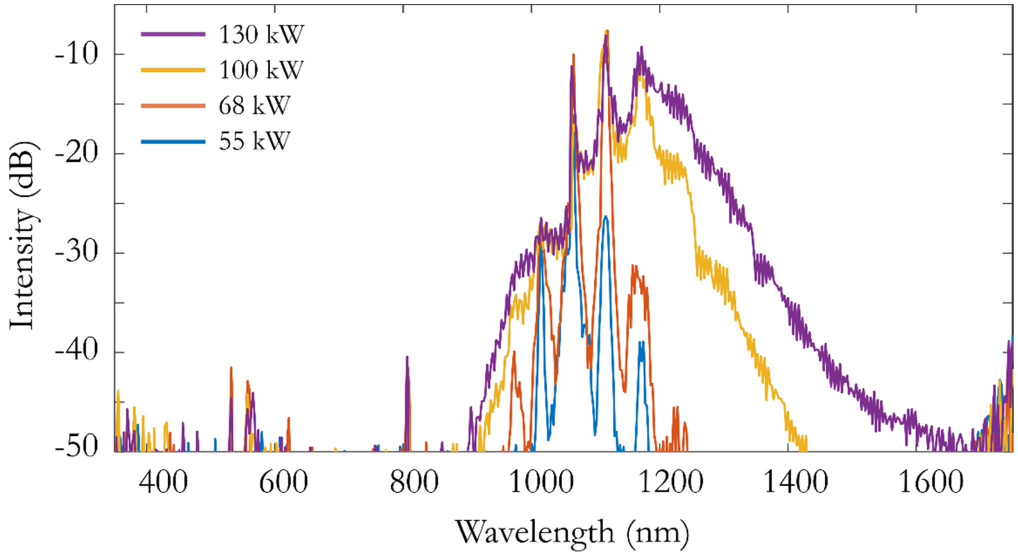

Supplementary Figure 4 | Output spectrum of a 1-m multimode fiber taper. The spectra are measured at the end of a 1-m tapered multimode fiber with the core radius changing from 25 to 10 µm. The power has raised from tens of kW to 180 kW, however, there is no sign of any sideband generation process.

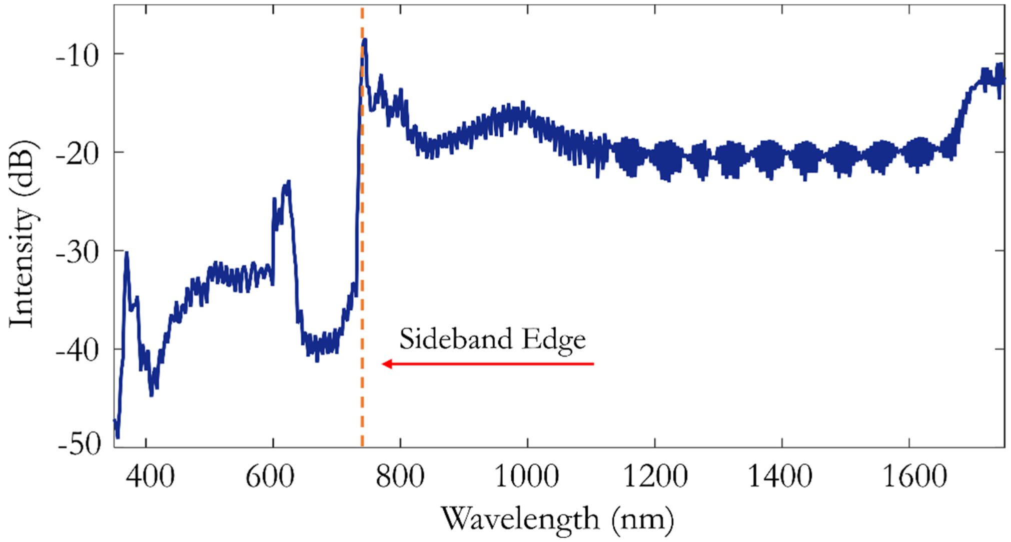

Supplementary Figure 5 | Output spectrum of a 3-m fiber taper. The spectrum is measured at the output of a 3-m long multimode fiber taper with a core radius changing from 40 to 20 µm. The sideband generation process has pushed the spectrum edge down to 750 nm.

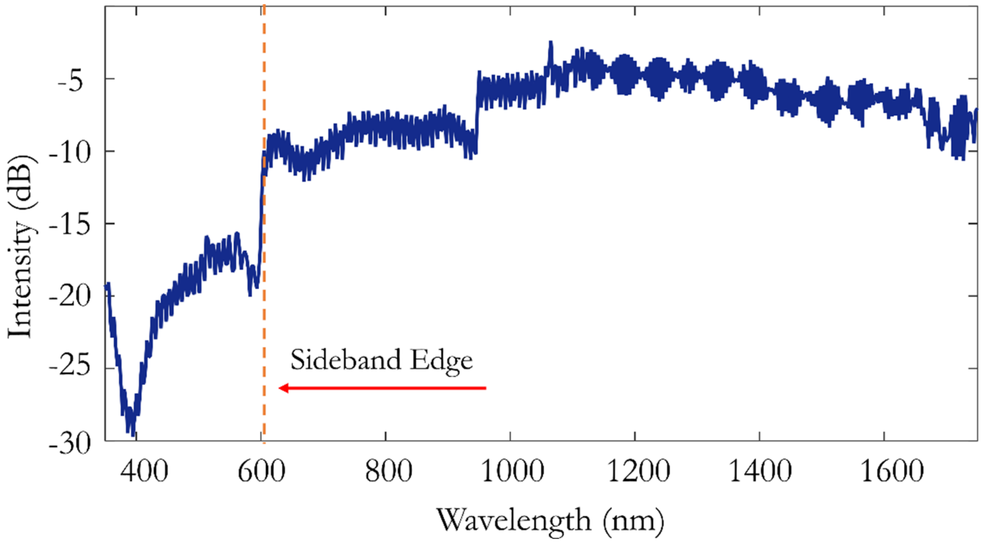

Supplementary Figure 6 | Output spectrum of a 5-m fiber taper. The spectrum is measured at the output of a 5-m long multimode fiber taper with a core radius changing from 40 to 10 µm. The sideband generation has pushed the spectrum edge to 600 nm and has filled the gap between 600 and 750 nm.
