# Supplementary material for: Accelerated nonlinear interactions in graded-index multimode fibers
Source: Nat Commun. 2019 Apr 9;10:1638. doi: 10.1038/s41467-019-09687-9 (PMC6456603; doi:10.1038/s41467-019-09687-9)
Supplement: Supplementary file 2 — Description of Additional Supplementary Files [file 41467_2019_9687_MOESM2_ESM.pdf]

**Description of Additional Supplementary Files**

File Name: Supplementary Movie 1
Description: Destabilization of solitons entering in the tapered section, showing energy flowing towards higher-order modes as they lose their quasi-Gaussian beam profiles.
